# Prospective evaluation of the Quality of Life after Brain Injury (QOLIBRI) score: minor differences in patients with major versus no or mild traumatic brain injury at one-year follow up

**DOI:** 10.1186/s12955-018-0966-z

**Published:** 2018-07-09

**Authors:** Konstantin Born, Felix Amsler, Thomas Gross

**Affiliations:** 1 Department of Traumatology, Cantonal Hospital Aarau, Tellstrasse, CH-5001 Aarau, Switzerland; 2 Basel, Switzerland; 3 Department of Traumatology, Cantonal Hospital Aarau, Tellstrasse, CH-5001 Aarau, Switzerland

**Keywords:** Quality of life after brain injury (QOLIBRI), Health-related quality of life (HRQoL), Outcome, Traumatic brain injury (TBI), Trauma, Longer-term, euro quality of life group health-related quality of life on five dimensions (EQ-5D), Medical outcomes study short Form-36 (SF-36), Trauma outcome profile (TOP)

## Abstract

**Background:**

The Quality of Life after Brain Injury (QOLIBRI) score was developed to assess disease-specific health-related quality of life (HRQoL) after traumatic brain injury (TBI). So far, validation studies on the QOLIBRI were only conducted in cohorts with traumatic brain injury. This study investigated the longer-term residuals in severely injured patients, focusing specifically on the possible impact of major TBI.

**Methods:**

In a prospective questionnaire investigation, 199 survivors with an injury severity score (ISS) > 15 participated in one-year follow-up. Patients who had sustained major TBI (abbreviated injury scale, AIS head > 2) were compared with patients who had no or only mild TBI (AIS head ≤ 2). Univariate analysis (ANOVA, Cohen’s kappa, Pearson’s r) and stepwise linear regression analysis (B with 95% CI, R, R^2^) were used.

**Results:**

The total QOLIBRI revealed no differences in one-year outcomes between patients with versus without major TBI (75 and 76, resp.; *p* = 0.68). With regard to the cognitive subscore, the group with major TBI demonstrated significantly more limitations than the one with no or mild TBI (*p* < 0.05). The AIS head correlated significantly with the cognitive dimension of the QOLIBRI (*r* = − 0.16; *p* < 0.05), but not with the mental components of the SF-36 or the TOP. In multivariate analysis, the influence of the severity of head injury (AIS head) on total QOLIBRI was weaker than that of injured extremities (R^2^ = 0.02; *p* < 0.05 vs. R^2^ = 0.04; *p* = 0.001) and equal to the QOLIBRI cognitive subscore (R^2^ = 0.03, *p* < 0.01 each).

**Conclusions:**

Given the unexpected result of similar mean QOLIBRI total score values and only minor differences in cognitive deficits following major trauma independently of whether patients sustained major brain injury or not, further studies should investigate whether the QOLIBRI actually has the discriminative capacity to detect specific residuals of major TBI. In effect, the score appears to indicate mental deficits following different types of severe trauma, which should be evaluated in more detail.

**Trial registration:**

NCT02165137; retrospectively registered 11 June 2014.

## Background

Given the increasing interest in the longer-term outcomes following severe trauma, several generic measures of subjective health status, such as the Short-Form Health Survey-36 (SF-36) or the EuroQoL five dimensions questionnaire (EQ-5D), as well as trauma related functional outcome instruments, such as the Trauma Outcome Profile (TOP) were evaluated in recent years [1]. Traumatic brain injury (TBI) accounts for an important percentage of severe trauma [2–4] and is reported depending partly on the exact definition of TBI [5, 6] and partly on the spectrum and severity of trauma chosen [7, 8]. In addition, the impact of TBI on the traumatized patient may be even more important than other body lesions both in monotrauma and multiple trauma [9, 10]. The newly developed Quality of Life after Brain Injury score (QOLIBRI) [11] was the first instrument designed to assess disease-specific health-related quality of life (HRQoL) following brain injury [12]. We therefore chose this score for our investigation, which still appears to be the most commonly used score for this topic, even though other instruments have been developed in the meantime [13–15]. Interestingly, work on the QOLIBRI is based fundamentally on original initiatives aimed at assessing non-physical residuals following trauma in general and developing a disease-specific HRQoL tool for multiple trauma [11, 16]. Longer-term follow-up studies in trauma patients both with and without TBI underscored the need to supplement the SF-36, for example, with a measure of cognitive function when evaluating outcome. Few differences were found with regard to scoring of cognitive function in phone interviews between TBI patients (matched according to grade on the Abbreviated Injury Scale, AIS) with and without additional orthopaedic injury [17]. Given the lack of knowledge in the literature on the degree to which cognitive deficits following trauma, whether independently of or dependent upon the TBI sustained, will be reliably identified by scores for HRQoL and functional outcome, we were interested in specifically investigating the QOLIBRI by assessing the longer-term course of patients who had suffered severe trauma (Injury Severity Score (ISS) > 15) and comparing it to other well established outcome scores. Currently, QOLIBRI validation studies have been undertaken only in cohorts with TBI, defined using the Glasgow Coma Scale (GCS), with case selection based on the International Classification of Diseases (ICD) and restricted to patients of working age [12, 18, 19]. In contrast, clinicians are accustomed to grading trauma severity according to the AIS and ISS independently of age. Literature searches did not reveal any investigations of the QOLIBRI that involved unselected, i.e. severely injured patients, including those without head injury, or evaluated possible correlations with trauma severity classified by AIS-grading.

Against this background we undertook a prospective trauma centre evaluation of the QOLIBRI with regard to the longer-term outcomes for the severely injured. The study cohort comprised consecutive patients who had sustained severe trauma to any body region, using the AIS to define the presence or absence of TBI and the severity of trauma. The investigation’s objective was to compare one-year outcomes in the form of QOLIBRI scores for patients with major versus no or only mild TBI by performing detailed analysis of the underlying demographic and trauma characteristics and in relation to established HRQoL and functional outcome instruments.

## Methods

The investigation took place in a dedicated trauma centre in Switzerland, serving a region of about 750,000 inhabitants. As part of a quality control project (NCT02165137) all major trauma patients (New Injury Severity Score, NISS ≥8) passing through the emergency department from 1.1.2011–31.12.2015 within 24 h of trauma were consecutively evaluated for this prospective investigation with a cross-sectional study design, approved by the local ethics committee. Hospital treatment guidelines followed international standards [20, 21]. This follow-up study included all survivors of trauma admitted to the emergency department of the hospital who were > 15 years of age at the time of the accident and who sustained severe trauma defined as an Injury Severity Score (ISS) > 15 [3, 22]. Major TBI was defined as a trauma severity of > 2 [23, 24] according to the Abbreviated Injury Scale (AIS) [25] of the head (version 2005, update 2008 of the TraumaRegister of the German Trauma Society). The no or mild TBI group contained all injured persons in the study cohort who did not sustain a trauma severity of > 2 according to the AIS head. AIS coding was executed according to the guidelines of the Association for the Advancement of Automotive Medicine (AAAM). For this evaluation of trauma cases all with a minimum ISS > 15, monotrauma was defined as an injury severity of > 3 according to the AIS in one body region and no injuries in other body regions (> 0) [4]. The term multiple trauma was used if at least two Abbreviated Injury Scale (AIS) regions were involved, and the ISS determined at the end of the hospital stay was 16 or above [26]. Given pilot evidence showing only a minimal impact of age on disease-specific HRQoL as measured by the QOLIBRI [27], and, in contrast to original validation studies of the QOLIBRI, we did not exclude retirees from this investigation, but controlled for age in multivariate analysis. Exclusion criteria for this survey were patients under the age of 16, those with an ISS ≤15, those deceased or presenting with a Glasgow Outcome Scale (GOS) [28] of 2 (persistent vegetative state) at hospital discharge or follow-up.

### Data management

Data management was executed by specifically trained study nurses who were not involved in the treatment of single cases. Injury severity was determined based on the maximum information available at the end of hospitalisation. The survival status of non-responders at the time of follow-up was controlled by contacting next of kin, family practitioners and local registry offices. Patients’ longer-term outcomes were assessed one year after trauma by a postal survey, complemented by phone interviews for missing or implausible answers undertaken by specifically trained study nurses. Standardised self-reporting questionnaires comprised a combination of validated quality of life (QoL) and functional scoring instruments with respect to outcome measurements. Written informed consent was obtained from study participants.

Demographic characteristics included age at time of injury (years) and gender (male/female).Injury-related variables were recorded by GCS [29], AIS, ISS, New Injury Severity Score (NISS) [30], Revised Injury Severity Classification(RISC) [31] and Simplified Acute Physiology Score (SAPS II) and involved predicting mortality [32]. Additionally, the Glasgow Outcome Scale (GOS) [28] post-injury at hospital discharge was determined. To differentiate the gradations of injuries to the brain and the rest of the body, we used AIS head and “NISS without AIS head”, subtracting AIS head squared from NISS.

The postal follow-up questionnaire one year after trauma included the following standard scores and subscores, all in their original forms and using validated translations where necessary: GOS, Euro Quality of Life Group [http://www.euroqol.org] health-related quality of life on five dimensions (EuroQoL; EQ-5D) and Visual Analogue Scale (EQ VAS) [33], medical outcomes study Short Form-36 (SF-36) [34], the Trauma Outcome Profile (TOP) [35, 36] and the QOLIBRI [18, 37]. With regard to mental or cognitive subscores, we used the original wording provided in the relevant publications as cited, but did not further discriminate between ‘mental’ or ‘cognitive’ in the context of this paper.

### Statistical analyses

In accordance with the major study objective one-year outcomes obtained with the QOLIBRI were compared for patients with major versus no or mild TBI. In more detailed analysis demographic and trauma characteristics were correlated with HRQoL and functional outcome instruments. Multivariate regression analysis was performed to detect any specific influences on the QOLIBRI and its mental subscore. Data are displayed as mean ± standard deviation (SD) for numeric variables. Numbers and percentages are given for nominal variables if not stated otherwise. All statistical tests are two-tailed and *p* < 0.05 was considered significant. Patients were entered into analysis if any follow-up data were received (*n* = 199). All correlations and their significance were tested parametrically and nonparametrically. Because there were only minimal differences in the correlations (r vs. rho) and the significances, and in order to present all variables in a comparable manner, especially in correlation analysis, nonparametric analyses are not shown. For univariate statistics, missing cases were excluded variable-wise. For multivariate analysis, missing values were replaced by the mean of the whole cohort.

Documented variables suspected or known from the literature to be possible factors associated with outcome were first analyzed by univariate analysis. ANOVA was used to compare group differences between non-respondents and respondents, between patients with no or mild TBI and major TBI and between subgroups of patients. To measure the agreement of categorized measures, Cohen’s kappa coefficient was used. In order not to lose too much statistical power due to small numbers of patients per cell, the subgroup of no or mild TBI patients was not further subdivided separately according to the AIS head, but rather the correlative and multivariate model was used to control for potential influences. Correlation testing was executed giving Pearson’s r.

Forward stepwise linear regression analysis to explain QOLIBRI total and QOLIBRI mental scales was performed by including all factors that were found to be significant in univariate analysis with the entry criterion *p* < 0.05 and a removal criterion *p* > 0.1. To exclude any potential impact of age this variable was included first in multivariable analysis after which all resulting associations were interpreted accordingly. Results are presented as B with 95% CI, R, R^2^ and *p*-values, additionally controlled for age and respecting change values.

Data were analyzed using IBM SPSS Statistics for Windows 24.0 (Armonk, NY: IBM Corp.).

## Results

**Fig. 1:**
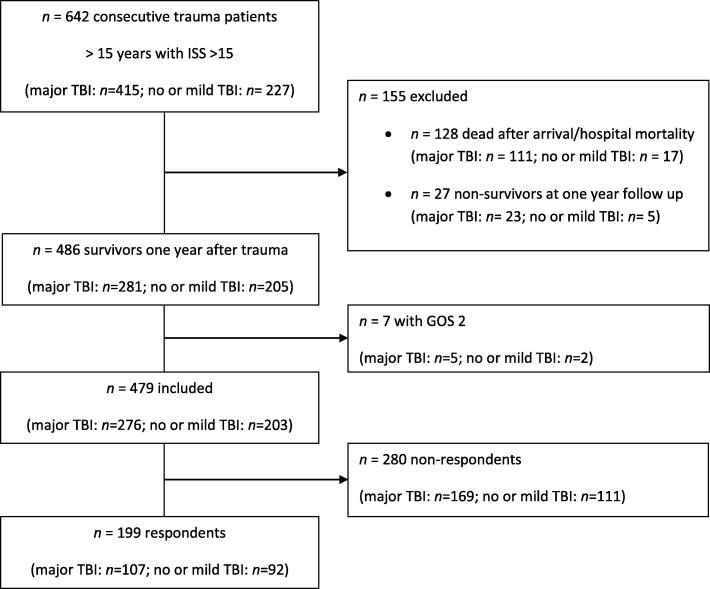
Patient inclusion flow chart

One hundred ninety-nine severely injured persons responded to the one-year follow-up, i.e. 41.5% of eligible persons (Fig. 1). 53.8% of investigated patients had sustained major TBI. The main patient and trauma characteristics did not differ between respondents and non-respondents (Table 1). Patients with major TBI differed from those without (48 cases with an AIS head = 0, 10 cases with an AIS head = 1 and 34 cases with an AIS head = 2) in most trauma specific characteristics, presenting with more severe trauma in the AIS 1 and 2 regions (head & neck and face) and less injured in all other body regions. No difference was found between groups regarding age, gender or overall ISS (Table 2).

**Table 1:** Comparison of respondents vs. non-respondents

	Non-respondents(*n* = 280)		Respondents(*n* = 199)		Comparison
	*n*	Mean + SD	*n*	Mean + SD	*p*
Age at time of injury (years)	280	54.31 ± 22.09	199	54.90 ± 18.49	0.757
GCS initial	278	12.93 ± 3.43	198	12.68 ± 3.80	0.451
GCS worst (within 24 h hours)	278	12.24 ± 4.17	198	12.05 ± 3.98	0.632
ISS	280	22.03 ± 6.94	199	22.01 ± 6.28	0.970
NISS	280	27.00 ± 9.78	199	27.26 ± 8.94	0.762
RISC (%)	280	13.41 ± 18.15	199	10.53 ± 13.38	0.058
SAPS II mort	142	26.59 ± 13.05	132	28.52 ± 10.95	0.189
AIS 1 head& neck	280	2.73 ± 1.67	199	2.54 ± 1.66	0.226
AIS 2 face	280	0.57 ± 1.01	199	0.56 ± 0.97	0.882
AIS 3 chest	280	1.61 ± 1.62	199	1.77 ± 1.57	0.272
AIS 4 abdomen	280	0.71 ± 1.21	199	0.83 ± 1.36	0.297
AIS 5 extremities	280	1.27 ± 1.34	199	1.39 ± 1.37	0.338
AIS 6 external lesions	280	0.38 ± 0.64	199	0.38 ± 0.58	0.904
AIS head	280	2.62 ± 1.71	199	2.45 ± 1.70	0.296
GOS (hospital discharge)	275	4.65 ± 0.58	196	4.69 ± 0.55	0.434
	*n*	*n* (%)	*n*	*n* (%)	*p*
Gender, female	280	89 (31.8%)	199	57 (28.6%)	0.463
Multiple trauma	280	220 (78.6%)	199	168 (84.4%)	0.108
High trauma energy	275	136 (49.5%)	199	113 (56.8%)	0.115
Lowest GCS 3–8	278	19 (17.6%)	198	38 (19.2%)	0.436
Lowest GCS 9–12		21 (7.6%)		20 (10.1%)	
Lowest GCS 13–15		208 (74.8%)		140 (70.7%)	

*GCS* Glasgow Coma Scale; *ISS* Injury Severity Score; *NISS* New Injury Severity Score; *RISC* Revised Injury Severity Classification; *SAPS II mort* expected simplified acute physiology score II mortality; *AIS (AIS 1: head & neck;* etc.*)* Abbreviated Injury Scale; *GOS* Glasgow Outcome Scale at the time of hospital discharge

**Table 2:** Patient and trauma characteristics of patients with major vs. no or mild TBI

	No or mild (AIS head 0–2) (*n* = 92)		Major TBI (AIS head 3–5) (*n* = 107)		Comparison
	*n*	Mean + SD	*n*	Mean + SD	*p*
Years since injury	92	1.08 ± 0.17	107	1.06 ± 0.16	0.481
Age at time of injury (years)	92	52.91 ± 18.02	107	56.61 ± 18.80	0.160
GCS initial	92	14.12 ± 2.17	106	11.42 ± 4.42	< 0.001
GCS worst (within 24 h hours)	92	13.84 ± 2.62	106	10.50 ± 5.04	< 0.001
ISS	92	21.47 ± 7.08	107	22.47 ± 5.50	0.264
NISS	92	24.46 ± 9.21	107	29.67 ± 7.98	< 0.001
RISC (%)	92	5.81 ± 8.64	107	14.58 ± 15.31	< 0.001
SAPS II mort	62	26.84 ± 10.46	70	30.00 ± 11.23	0.098
AIS 1 head & neck	92	1.03 ± 1.09	107	3.83 ± 0.69	< 0.001
AIS 2 face	92	0.24 ± 0.70	107	0.83 ± 1.08	< 0.001
AIS 3 chest	92	2.52 ± 1.39	107	1.13 ± 1.44	< 0.001
AIS 4 abdomen	92	1.55 ± 1.57	107	0.21 ± 0.70	< 0.001
AIS 5 extremities	92	2.02 ± 1.34	107	0.85 ± 1.16	< 0.001
AIS 6 external lesions	92	0.43 ± 0.67	107	0.34 ± 0.49	0.235
AIS head	92	0.85 ± 0.94	107	3.83 ± 0.69	< 0.001
GOS (hospital discharge)	92	4.78 ± 0.47	104	4.61 ± 0.60	0.023
	*n*	*n* (%)	*n*	*n* (%)	*p*
Gender, female	92	27 (29.3%)	107	30 (28%)	0.839
Multiple trauma	92	85 (92.4%)	107	83 (77.6%)	0.004
Trauma energy high	92	65 (70.7%)	107	48 (44.9%)	< 0.001
Lowest GCS 3–8	92	5 (5.4%)	106	33 (31.3%)	< 0.001
Lowest GCS 9–12		7 (7.6%)		13 (12.3%)	
Lowest GCS 13–15		80 (87.0%)		60 (56.6%)	

No or mild TBI: no or mild traumatic brain injury (AIS head ≤ 2); *major TBI* major traumatic brain injury (AIS head > 2); *GCS* Glasgow Coma Scale; *ISS* Injury Severity Score; *NISS* New Injury Severity Score; *RISC* Revised Injury Severity Classification; *SAPS II mort* expected simplified acute physiology score II mortality; AIS (AIS 1: head & neck; etc.): Abbreviated Injury Scale; *GOS* Glasgow Outcome Scale at the time of hospital discharge

### No or mild TBI vs. major TBI

One year after injury, the mean total QOLIBRI for all study participants was 75.5 ± 21.2. Severely injured with major TBI presented with a mean total QOLIBRI of 74.9, comparable to patients with no or mild TBI (76.1; Table 3). With regard to the cognitive subscore the group with major TBI demonstrated significantly more limitations than did no or mild TBI patients (*p* = 0.038). On the other hand, patients with major TBI demonstrated significantly less limitations in the EuroQoL (*p* = 0.017), the physical component of the SF-36 (*p* = 0.015), and the TOP (*p* = 0.035) but not for the GOS (*p* = 0.310).

**Table 3:** Outcome scores of patients with major vs. no or mild TBI

	No or mild TBI (*n* = 92)		Major TBI (*n* = 107)		Comparison
	*n*	Mean + SD	*n*	Mean + SD	*p*
Q cognition	92	78.10 ± 23.50	106	70.49 ± 27.20	0.038
Q self	92	72.46 ± 23.11	106	71.66 ± 23.54	0.812
Q daily life & autonomy	92	75.95 ± 25.09	106	76.6 ± 26.29	0.861
Q social relationships	92	75.56 ± 23.07	105	77.91 ± 20.23	0.448
Q emotions	92	81.85 ± 22.34	106	84.86 ± 21.49	0.336
Q physical problems	92	71.07 ± 25.58	106	76.47 ± 23.35	0.122
QOLIBRI total score	92	76.14 ± 20.77	106	74.87 ± 21.65	0.676
SF-36 physical	86	43.46 ± 10.68	100	47.33 ± 10.76	0.015
SF-36 mental	86	47.73 ± 14.25	100	44.98 ± 14.08	0.188
EuroQoL	92	0.68 ± 0.23	106	0.75 ± 0.22	0.017
GOS (one-year follow-up)	92	4.67 ± 0.56	105	4.75 ± 0.52	0.310
TOP mental functioning	92	69.29 ± 27.26	107	65.63 ± 27.35	0.347
TOP physical component	92	81.68 ± 18.35	105	87.03 ± 16.91	0.035
TOP mental component	92	78.20 ± 21.27	106	79.09 ± 19.94	0.762

No or mild TBI, no or mild traumatic brain injury (AIS head ≤ 2); major TBI, major traumatic brain injury (AIS head > 2); SF-36: trauma medical outcomes study Short Form-36 (physical and mental sum component); *EuroQoL* Euro Quality of Life; *GOS* Glasgow Outcome Scale at one year follow-up; *TOP* Trauma Outcome Profile and dimensions; *Q: QOLIBRI*, Quality of Life after Brain Injury

Comparing major TBI with no or mild TBI patients 20.8% (*n* = 22) and 18.5% (*n* = 17), respectively, presented with a total QOLIBRI of < 60 (*p* = 0.690) and were defined as having an impaired HRQoL according to Wilson [38]. 27.0% (*n* = 27) achieved an SF-36 mental (MCS) < 40 (*p* = 0.629) and were considered conspicuous. In 15.7% of cases both the QOLIBRI and the SF-36 MCS scores were impaired (Kappa 0.58; *p* < 0.001).

### Comparison of combined criteria (TBI and GCS)

If groups of major TBI and no or mildTBI were additionally controlled for the worst GCS within the first 24 h hours following trauma (Table 4), heterogeneous results were found. Lower GCS was accompanied by lower QOLIBRI measures in both groups: For no or mild TBI patients it was the subgroup with a GCS < 8 (severe) that achieved the worst outcome in QOLIBRI cognition, physical and total, whereas for patients with major TBI this was the case for the subgroup with a GCS 9–12 (moderate). The same pattern was found for SF-36 physical, EuroQol, GOS and TOP mental function (all *p* < 0.05).

**Table 4:** Outcome scorings of patients with major vs. no or mild TBI including post-injury GCS-status

	AIS head 0–2 GCS 3–8 (*n* = 5)		AIS head 0–2 GCS 9–12 (*n* = 7)		AIS head 0–2 GCS13–15 (*n* = 80)		AIS head 3–5 GCS 3–8 (*n* = 33)		AIS head 3–5 GCS 9–12 (*n* = 13)		AIS head 3–5 GCS 13–15 (*n* = 60)		Comparison
	*n*	Mean + SD	*n*	Mean + SD	*n*	Mean + SD	*n*	Mean + SD	*n*	Mean + SD	*n*	Mean + SD	*p*
Q cognition	5	55.71 ± 37.93	7	76.53 ± 19.44	80	79.63 ± 22.37	32	71.54 ± 26.84	13	59.34 ± 28.22	60	71.85 ± 27.04	0.043
Q self	5	57.86 ± 27.94	7	66.84 ± 21.79	80	73.86 ± 22.84	32	71.21 ± 25.85	13	61.54 ± 17.57	60	73.63 ± 23.06	0.342
Q daily life & autonomy	5	45.71 ± 36.63	7	65.48 ± 25.03	80	78.76 ± 23.11	32	73.40 ± 25.53	13	57.42 ± 29.41	60	82.06 ± 24.19	0.001
Q social relationships	5	52.50 ± 38.93	7	76.79 ± 29.94	80	76.90 ± 20.81	31	75.35 ± 19.99	13	70.71 ± 14.91	60	80.49 ± 21.12	0.097
Q emotions	5	68.00 ± 23.61	7	82.86 ± 24.47	80	82.63 ± 22.10	32	86.09 ± 17.59	13	78.85 ± 28.30	60	85.25 ± 21.99	0.547
Q physical problems	5	45.00 ± 22.08	7	63.57 ± 17.49	80	73.36 ± 25.55	32	77.34 ± 19.96	13	61.54 ± 30.37	60	78.94 ± 22.63	0.010
QOLIBRI total score	5	54.27 ± 31.18	7	72.50 ± 19.62	80	77.82 ± 19.61	32	74.44 ± 21.15	13	63.34 ± 21.30	60	77.19 ± 21.50	0.052
SF-36 physical	5	36.52 ± 13.21	7	36.83 ± 12.98	74	44.56 ± 10.04	31	45.51 ± 12.64	13	41.38 ± 12.52	55	49.58 ± 8.46	0.002
SF-36 mental	5	36.36 ± 23.80	7	47.73 ± 13.00	74	48.50 ± 13.49	31	43.81 ± 14.30	13	41.27 ± 15.00	55	46.26 ± 13.80	0.230
EuroQoL	5	0.52 ± 0.09	7	0.56 ± 0.23	80	0.70 ± 0.23	33	0.74 ± 0.22	13	0.67 ± 0.24	59	0.78 ± 0.21	0.018
GOS (one year follow-up)	5	4.20 ± 0.84	7	4.43 ± 0.54	80	4.73 ± 0.53	31	4.71 ± 0.59	13	4.46 ± 0.66	60	4.83 ± 0.42	0.027
TOP mental functioning	5	47.73 ± 34.59	7	56.57 ± 28.25	80	71.75 ± 26.17	33	67.11 ± 25.92	13	46.05 ± 24.41	60	68.51 ± 27.31	0.015
TOP number of conspicuous subscores	5	6.00 ± 2.74	7	4.43 ± 2.88	79	3.20 ± 3.12	33	2.64 ± 2.40	13	3.85 ± 3.31	59	2.97 ± 2.80	0.155
TOP physical component	5	72.02 ± 9.53	7	77.09 ± 23.70	80	82.69 ± 18.21	32	86.84 ± 14.07	13	80.01 ± 28.08	59	88.47 ± 15.10	0.114
TOP mental component	5	60.10 ± 24.16	7	76.21 ± 19.05	79	79.52 ± 21.00	33	80.28 ± 18.38	13	71.30 ± 24.69	59	79.79 ± 19.63	0.271

*AIS* Abbreviated Injury Scale; *GCS* Glasgow Coma Scale; *SF-36* trauma medical outcomes study Short Form-36 (physical and mental sum component); *EuroQoL* Euro Quality of Life; *GOS* Glasgow Outcome Scale at one year follow-up; *TOP* Trauma Outcome Profile and dimensions; *Q*: QOLIBRI, Quality of Life after Brain Injury

### Correlation and regression analysis

Univariate correlation testing of outcome variables with patient and trauma characteristics (Table 5) showed significant but low interrelation values whereby a maximum association (*r* = − 0.39; *p* < 0.001) was found on the EuroQol for the AIS 5 region (extremities). The total QOLIBRI also revealed its highest correlation for the AIS 5 region (*r* = − 0.18; *p* < 0.05), whereas the cognitive dimension of the QOLIRBI correlated best with AIS head (*r* = − 0.16; *p* < 0.05). In contrast, the mental components of the SF-36 and the TOP did not show any significant correlation with AIS head. All investigated outcome scores showed only a low correlation in univariate analysis with age, including the total QOLIBRI (*r* = − 0.12; *p* = n.s.).

**Table 5:** Univariate correlation of outcome scores with patient and trauma characteristics

Pearson r	Age	Gender, female	ISS	NISS	NISS without AIS head squared	AIS head	AIS 1 head & neck	AIS 2 face	AIS 3 chest	AIS 4 abdomen	AIS 5 extremities	AIS 6 external lesions	RISC (%)	GCS initial	GCS lowest	High trauma energy	GOS	Discharge to a rehabilitation hospital
Q cognition	−0.16*	0.06	−0.11	−0.06	0.06	−0.16*	−0.15*	−0.10	0.01	0.09	−0.08	−0.06	−0.19**	0.11	0.13	0.00	0.14	−0.19**
Q self	−0.03	−0.04	−0.11	−0.05	−0.04	−0.03	−0.02	−0.13	−0.06	0.04	−0.16*	−0.02	−0.08	0.07	0.10	−0.07	0.12	−0.15*
Q daily life & autonomy	−0.11	0.03	−0.17*	−0.12	−0.13	−0.01	−0.01	−0.09	−0.06	0.02	−0.25***	−0.08	−0.14	0.14	0.18*	−0.14	0.27***	−0.23***
Q socia lrelationships	−0.03	− 0.01	−0.08	−0.07	−0.08	−0.01	0.02	−0.04	−0.12	−0.03	− 0.14	−0.04	−0.04	0.09	0.12	−0.17*	0.12	−0.17*
Q emotions	0.00	−0.02	−0.09	0.00	−0.03	0.01	0.03	−0.13	−0.06	0.00	−0.16*	−0.20**	−0.01	−0.03	0.01	−0.05	0.00	−0.15*
Q physical problems	−0.17*	0.02	−0.11	−0.05	−0.14	0.07	0.09	−0.08	−0.08	0.00	−0.28***	−0.17*	−0.11	0.03	0.06	−0.12	0.20**	−0.23***
QOLIBRI total score	−0.12	0.02	−0.13	−0.07	−0.04	−0.06	−0.05	−0.11	−0.05	0.04	−0.18*	−0.10	−0.14	0.09	0.13	−0.08	0.17*	−0.22**
SF-36 physical	−0.19*	0.01	−0.25***	−0.07	−0.24***	0.19**	0.17*	0.01	−0.19**	−0.08	−0.32***	−0.17*	−0.11	0.06	0.08	−0.15*	0.37***	−0.26***
SF-36 mental	0.01	−0.07	−0.04	−0.12	0.01	−0.14	−0.12	−0.14	0.06	0.12	−0.04	−0.01	−0.16*	0.11	0.15*	0.00	0.12	−0.05
EuroQoL	−0.10	0.00	−0.13	−0.02	−0.19**	0.16*	0.17*	−0.09	−0.14	−0.06	−0.39***	−0.22**	−0.02	0.03	0.05	−0.18**	0.23***	−0.19**
GOS (one-year follow-up)	−0.16*	0.05	−0.20**	−0.17*	−0.25***	0.09	0.10	−0.07	−0.05	−0.03	−0.26***	−0.11	−0.12	0.04	0.11	−0.12	0.37***	−0.22**
TOP mental functioning	−0.06	0.00	−0.16*	−0.08	−0.01	−0.09	−0.07	−0.14	−0.01	0.04	−0.12	−0.07	−0.15*	0.07	0.11	−0.04	0.22**	−0.22**
TOP number of conspicuous subscores	0.09	0.07	0.19**	0.08	0.15*	−0.06	−0.07	0.10	0.10	0.06	0.25***	0.15*	0.08	0.02	−0.01	0.09	−0.11	0.10
TOP physical component	−0.03	0.00	−0.20**	−0.07	−0.20**	0.14	0.13	−0.07	−0.12	−0.06	−0.31***	−0.18*	0.02	0.01	0.02	−0.09	0.16*	−0.13
TOP mental component	0.04	−0.03	−0.13	−0.08	−0.08	−0.01	0.01	−0.10	−0.01	−0.03	−0.17*	−0.14	−0.05	0.01	0.05	−0.08	0.08	−0.13

*SF-36* trauma medical outcomes study Short Form-36 (physical and mental sum component), *EuroQoL* Euro Quality of Life, *GOS* Glasgow outcome score at one year follow-up, *TOP* Trauma Outcome Profile and dimensions, *Q* QOLIBRI (Quality of Life after Brain Injury), *ISS* Injury Severity Score, *NISS* New Injury Severity Score, *AIS* Abbreviated Injury Scale, *RISC* Revised Injury Severity Classification; *GCS*: Glasgow Coma Scale; *GOS*: Glasgow Outcome Scale at one year follow-up; Trauma energy: high vs. low

* *p* < 0.05; ** *p* < 0.01; *** *p* < 0.001

Comparison of outcome variables with each other is shown in Table 6. The total QOLIBRI demonstrated the highest association with the mental component of the TOP (*r* = 0.83; *p* < 0.001) and lowest with the physical subscore of the SF-36 (*r* = 0.40; *p* < 0.001). The cognitive dimension of the QOLIBRI correlated best with mental functioning of the TOP (*r* = 0.71; *p* < 0.001).

**Table 6:** Univariate correlation of outcome scores with each other

Pearson r	Q cognition	Q self	Q daily life & autonomy	Q social relationships	Q emotions	Q physica lproblems	QOLIBRI total score	SF-36 physical	SF-36 mental	EuroQoL	GOS (one-year follow-up)	TOPQ: mental functioning	TOPQ: number of notable subscores	TOP physical component	TOP mental component
Q cognition	1	0.79***	0.76***	0.66***	0.63***	0.57***	0.92***	0.31***	0.60***	0.56***	0.46***	0.71***	−0.65***	0.53***	0.70***
Q self	0.79***	1	0.79***	0.78***	0.72***	0.60***	0.91***	0.31***	0.68***	0.60***	0.45***	0.68***	−0.71***	0.59***	0.76***
Q daily life & autonomy	0.76***	0.79***	1	0.76***	0.69***	0.76***	0.91***	0.47***	0.67***	0.72***	0.64***	0.69***	−0.77***	0.70***	0.76***
Q social relationships	0.66***	0.78***	0.76***	1	0.66***	0.56***	0.83***	0.23**	0.65***	0.54***	0.39***	0.56***	−0.62***	0.50***	0.67***
Q emotions	0.63***	0.72***	0.69***	0.66***	1	0.71***	0.81***	0.26***	0.71***	0.63***	0.51***	0.62***	−0.75***	0.67***	0.82***
Q physical problems	0.57***	0.60***	0.76***	0.56***	0.71***	1	0.77***	0.58***	0.55***	0.81***	0.73***	0.63***	−0.74***	0.76***	0.69***
QOLIBRI total score	0.92***	0.91***	0.91***	0.83***	0.81***	0.77***	1	0.40***	0.73***	0.72***	0.59***	0.76***	−0.80***	0.69***	0.83***
SF-36 physical	0.31***	0.31***	0.47***	0.23**	0.26***	0.58***	0.40***	1	0.18*	0.65***	0.52***	0.37***	−0.46***	0.53***	0.28***
SF-36 mental	0.60***	0.68***	0.67***	0.65***	0.71***	0.55***	0.73***	0.18*	1	0.52***	0.42***	0.66***	−0.75***	0.50***	0.84***
EuroQoL	0.56***	0.60***	0.72***	0.54***	0.63***	0.81***	0.72***	0.65***	0.52***	1	0.69***	0.60***	−0.74***	0.78***	0.69***
GOS (one-year follow-up)	0.46***	0.45***	0.64***	0.39***	0.51***	0.73***	0.59***	0.52***	0.42***	0.69***	1	0.52***	−0.56***	0.62***	0.53***
TOP: mental functioning	0.71***	0.68***	0.69***	0.56***	0.62***	0.63***	0.76***	0.37***	0.66***	0.60***	0.52***	1	−0.77***	0.57***	0.83***
TOP: number of conspicuous subscores	−0.65***	−0.71***	−0.77***	−0.62***	−0.75***	−0.74***	−0.80***	−0.46***	−0.75***	−0.74***	−0.56***	−0.77***	1	−0.78***	−0.89***
TOP physical component	0.53***	0.59***	0.70***	0.50***	0.67***	0.76***	0.69***	0.53***	0.50***	0.78***	0.62***	0.57***	−0.78***	1	0.70***
TOP mental component	0.70***	0.76***	0.76***	0.67***	0.82***	0.69***	0.83***	0.28***	0.84***	0.69***	0.53***	0.83***	−0.89***	0.70***	1

*SF-36* Trauma medical outcomes study Short Form-36 (physical and mental sum component), *EuroQoL* Euro Quality of Life, *GOS* Glasgow Outcome Scale at one year follow-up, *TOP* Trauma outcome profile and dimensions, *Q* QOLIBRI, Quality of Life after Brain Injury

* *p* < 0.05; ** *p* < 0.01; *** *p* < 0.001

Following multivariate regression analysis, the total QOLIBRI demonstrated low associations with trauma characteristics, best associations with the AIS 5 region (R^2^ = 0.04; *p* = 0.001) followed by the AIS head region (R^2^ = 0.03; *p* = 0.024), revealing a variance of 6% (R^2^ controlled for age; Table 7). The cognitive subscore of the QOLIBRI correlated equally with the AIS head (R^2^ = 0.03; *p* = 0.002) and extremities region (R^2^ = 0.03; *p* = 0.007).

**Table 7:** Multivariate regression analysis in relation to QOLIBRI total and cognition 1 year after trauma

Model	Variable	B	95% CI lower - upper		Beta	*p*	R	R^2^	R^2^ controlled for age	R^2^ change	F change	*p* change
	Dependent: QOLIBRI total											
	(Constant)	94.94	83.46	106.42		< 0.001						
1	Age at time of injury	−0.15	−0.31	0.01	−0.13	0.058	0.12	0.01		0.01	2.67	0.104
2	AIS 5 (extremities)	−4.17	−6.53	−1.82	−0.27	0.001	0.22	0.05	0.04	0.04	7.48	0.007
3	AIS head	−2.20	−4.09	−0.30	−0.18	0.024	0.27	0.07	0.06	0.02	5.21	0.024
	Dependent: QOLIBRI cognition											
	(Constant)	101.29	87.41	115.18		< 0.001						
1	Age at time of injury	−0.23	−0.42	−0.04	−0.17	0.015	0.15	0.02		0.025	5.14	0.024
2	AIS head	−3.71	−6.00	−1.41	−0.25	0.002	0.22	0.05	0.03	0.03	4.69	0.032
3	AIS 5 (extremities)	−3.90	−6.75	−1.06	−0.21	0.007	0.29	0.08	0.06	0.03	7.31	0.007

*QOLIBRI*: Quality of Life after Brain Injury; *AIS*: Abbreviated Injury Scale; *B*: non standardized beta (slope); *CI*: confidence interval; *Beta*: standardized beta (strength of the relationship), *R*: correlation coefficient; *R*^*2*^: explained variance; change: change of the additional variable entered into the model

## Discussion

To our knowledge, this is the first evaluation of the QOLIBRI with regard to one-year outcome in a cohort of severely injured patients that includes both major and no or only mild TBI. We found two unexpected major results:

1.), The total QOLIBRI did not correlate at all and the cognitive subscore correlated only poorly with major TBI in this investigation. 2.) The cognitive dimension of the QOLIBRI correlated weakly, but better with TBI than the other mental scales of HRQoL or functional outcome scores tested.

From a historical point of view, although von Steinbüchel et al. in their original evaluation studies correctly describe the broad spectrum of HRQoL to be measured by the QOLIBRI [11, 12, 18, 19], they and subsequent authors almost univocally argue for its use in TBI only. This is already indicated by the fact that its name includes *‘brain injury’*. This conclusion is even more astonishing as the founding consensus group explicitly cited the original initiatives, e.g. research by Neugebauer in the 90s, as aiming to develop a disease-specific HRQoL tool for multiple trauma [11]. Their efforts to assess non-physical residuals following trauma resulted in the development of scores such as the QOLIBRI. Reviewing the literature on the QOLIBRI to date, all subsequent studies have focused on TBI cohorts only. Beginning with the first validation studies published by von Steinbüchel et al. [12], the QOLIBRI remained an HRQoL instrument propagated only for traumatic brain injury, even though many of the score questions clearly indicate that a broader spectrum is to be covered.

This prospective investigation is the first to identify in a comparative manner deficits in a mixed cohort of severe trauma patients (ISS > 15) by implementation of the QOLIBRI as a standard questionnaire and including other validated scores on HRQoL and functional outcome such as the GOS, EuroQoL, SF-36 or the TOP for further evaluation.

Ad 1.), In effect, the *total QOLIBRI* did not discriminate between patients with and those without major TBI in our unselected cohort of severely injured persons (ISS > 15). No significant correlation could be found between the QOLIBRI and any measures of injury except for injuries of the extremities. Even additionally screening for TBI with the GCS did not (relevantly) improve the capacity of the QOLIBRI to detect specific residuals of TBI, given the finding of a comparable number of patients with no or mild TBI and depressed QOLIBRI values, independently of GCS-stratification. The GCS did not add significant information in multivariate analysis either.

Original validation studies report an *average total QOLIBRI score in TBI patients* of about 65 one year following trauma [11, 12, 18, 19]. A few studies showed mean values of about 5 points lower [39] or higher, even if the complete cohort under evaluation was limited to severe TBI only (defined as a GCS < 9) [40]. The mean QOLIBRI in our study was found to be 75, independently of whether patients sustained major TBI or not, with lowest values in patients with no or mild TBI. Wilson et al. suggested a grading for recovery after TBI for the QOLIBRI by stratification for the GOSE, whereby an average score of 76 meant good recovery, 62 moderate disability and 56 severe disability [38].Following this stratification, our patients presented with good recovery on average at the one-year follow-up. To explain this difference between the literature and our data, further comparative analysis of study cohorts and procedures in the literature has to be undertaken. Surprisingly, to date, apart from the isolated exception found in the publication by Soberg et al. [41], all investigations on the QOLIBRI only gave a precise definition of their study cohorts with regard to TBI, but not regarding possible additional body injuries or overall trauma severity of patients [11, 12, 18, 19, 38, 42]. Almost all investigations of the QOLIBRI selected their TBI patients based on the ICD-classification and defined the severity of TBI by the worst GCS within 24 h following trauma. Therefore, most studies report on cohorts that are comprised to about 55–60% of patients with a GCS < 8 or investigate severe TBI (GCS < 9) only [40, 41]. In contrast and similar to other trauma centre evaluations [4, 6], our definition of TBI was based on the prospective consecutive assessment of unselected trauma patients with trauma severity graded according to the AIS. Our study cohort comprised 19% patients with a GCS < 8, i.e. 31% of patients in the major TBI-group. At first glance, this important difference in the percentage of severe TBI patients identified by GCS (3–8) in our cohort compared to previous studies might explain the difference in outcomes as stated above and quantified with the QOLIBRI. Nevertheless, the analysis of only severe TBI-cases with a GCS 3–8 in our investigation (as used by QOLIBRI validation groups) did reveal a mean total QOLIBRI of 74, which was comparable to the whole study group and/or unselected TBI-patients in terms of GCS. In our study cohort the lowest total QOLIBRI values for patients with a GCS 3–8 (mean 54) were found in the small subgroup of no or mild TBI (*n* = 5), i.e. for patients with an AIS head 0–2. The literature regarding a possible association between the severity of TBI and resulting HRQoL appears conflicting with both better and worse outcomes reported for more severe trauma [43, 44]. As in our analysis, most international studies reported no association of the QOLIBRI with the GCS, with some exceptions such as the latest Finnish evaluation of TBI patients undergoing intensive residential rehabilitation that reported a low negative correlation (Spearman *r* = − 0.21) [39]. One possible explanation for such divergent findings may be the selection of the study samples under investigation. For example, in a large Trauma Audit Research Network database analysis of over 25,000 patients with isolated TBI (AIS head > 2) for an equivalent severity of intracranial injury (defined according to the AIS), GCS was found to be higher in older patients than in young ones, an observation unlikely to be explained by differences in mechanism of injury or types of intracranial injury according to the analysis of authors [45]. In addition, a general lack of standardization in assessment and reporting of the GCS was reported in recent surveys and reviews [46, 47] and may be, at least partially, responsible for the observed variation of outcome data.

With regard to the severity of head injury (AIS), comparison with a simpler outcome score such as the EuroQoL in our investigation surprisingly showed correlation values not inferior to the QOLIBRI cognition (Pearson *r* = 0.16). In addition, the magnitude of the correlations for the cognitive QOLIBRI with the AIS of the head were not higher than for the total QOLIBRI with the AIS of the extremities, underlining the finding that the QOLIBRI is not injury specific. Interestingly, higher correlations (r about 0.4) were found for the EuroQoL regarding the grade of injury of the extremities. Overall, more somatic oriented outcome (sub-) scores correlated better with somatic lesions than did the QOLIBRI and its subscores or the mental and cognitive dimensions of the SF-36 or the TOP with brain injury. According to the literature the SF-36 may not be sensitive enough to detect key issues in patients with a TBI, such as cognitive dysfunction, severe physical restrictions or patients with psychological problems. In consequence, to assess the sequelae of TBI the use of both a generic (e.g. the SF-36) and a disease-specific measure (e.g. the QOLIBRI) of HRQoL is recommended [10, 14], even though no standardised interpretation aids for such a combined use are yet available. One example is the POLO chart, representing a battery of scores developed for the multiply injured including TBI and comprising the GOS, the EQ-5D, the SF-36 and the TOP [36, 48]. Unfortunately, such extensive scoring appears difficult to handle for the standard evaluation of single patients, even though the trauma specific TOP in the few studies published to date appeared to be a reliable and well discriminating score, covering both relevant general dimensions of HRQoL and trauma-specific aspects of longer-term outcome [36, 49]. Currently, no reports on any comparison of the TOP with the QOLIBRI are available, the present investigation being the first to compare both scores in a trauma centre setting. With regard to the clinically relevant question as to which patients are identified as conspicuous based on their boundary score values and, therefore, needing further individual examination or even therapy, we found that in 31% one, i.e. either the SF-36 or the QOLIBRI, and in 16% both indicated an impaired HRQoL. Such findings additionally support the conclusion that scoring for detection primarily of non-somatic residuals following severe trauma still has to be improved [14]. A recent detailed correlation analysis of QOLIBRI and SF-36 in the original international validation cohort of the QOLIBRI demonstrated a higher discriminative power of the QOLIBRI and all its subscales in comparison to the SF-36. At the same time, the informative value of subscales did differentiate between moderate and good recovery categories [42].

Ad 2.) The *cognitive component* was the only QOLIBRI-score that differentiated between patients with versus without major TBI in univariate analysis. Recent studies reported that HRQoL and recovery patterns differ for mild, moderate and severe TBI [50] and, for the QOLIBRI, it was even shown that severe TBI patients may report better cognitive functioning on the QOLIBRI subscale than did mild TBI patients [51]. Surprisingly for a brain injury score, in multivariate analysis of our data the cognitive QOLIBRI subscore was found to be weakly, but equally, associated (R^2^ = 0.03) with both head trauma and injuries to other body regions (each classified according to the AIS). Nevertheless, in comparison with the mental subscales of the TOP and the SF-36, we found the cognitive QOLIBRI to be the only one to correlate, weakly, but still significantly with the AIS of the head. These findings provide further evidence that the cognitive QOLIBRI indeed elicits specific information on the mental outcome of patients. But, in contrast to the recommendations for its use only in TBI [14], the information on mental residuals following trauma found in our investigation was identified equally for patients with and without major TBI. In our study the cognitive component of the QOLIBRI showed the best association with the mental components of the TOP (*r* = 0.70), followed by the mental sum component of the SF-36 (*r* = 0.60). The association of the cognitive QOLIBRI with the physical sum component of the SF-36 was much lower (*r* = 0.31) – a finding that appears logical. Even though, for example, in the Australian validation study of the QOLIBRI [52], as part of the international QOLIBRI project using the identical inclusion criteria to those of von Steinbüchel et al. [18, 19], the cognitive QOLIBRI correlated less with the mental sum component of the SF-36 (*r* = 0.44), apparently having more in common with the physical sum component of the SF-36 (*r* = 0.53). This finding would not be expected for a TBI-specific score but, unfortunately, this discrepancy was not discussed further by the authors. One possible explanation for this result could be that only TBI-patients were included in their study.

Originally, von Steinbüchel et al. [18] stated that the items and therefore the total QOLIBRI score predominantly concentrate on emotional, cognitive, and psychosocial aspects and to a lesser extent on physical changes. The authors concluded that the questionnaire thus measures satisfaction and distress in areas of life typically affected by brain injury. Given our findings in unselected patients following severe trauma of all body regions, we are of the opinion that *the use of the QOLIBRI* should not be restricted to TBI patients only, but should also be undertaken in more severe non-TBI patients for whom such deficits are rarely expected and therefore not screened for on a routine basis. We are aware that such argumentation opposes the mainstream and would involve shifting focus to the development of defect-specific HRQoL scores that would also apply to TBI [14]. In agreement with investigators such as Dijkers [53] we are of the opinion that developing modules that quantify quality of life in specific functional areas that are underserved given the generic quality of life measures such as cognitive functioning may be a very reasonable approach. Due to the fact that no reports on the use of the QOLIBRI in no or mild TBI patients have been published so far, the pilot findings in this field as presented here have to be carefully weighed up and await further evaluation in cohorts other than ours.

### Limitations

The results presented here are limited to the single-centre character of this prospective study in survivors of severe trauma, graded as an ISS > 15 and with TBI defined according to the AIS head. At first sight a response rate of 42% at one year follow-up appears to be low, but it is well comparable with other reports for extended follow-up controls in the severely injured that include a bundle of standardised outcome instruments [10, 41, 49, 54, 55]. In addition, the characteristics of non-respondents did not differ from respondents, especially with regard to rate and grading of TBI as well as overall trauma severity. Given the low rate of patients with a GCS < 9, our data for the various subgroups have to be interpreted with caution. Overall results of this European trauma centre investigation should be fairly well representative of a consecutive cohort of severely injured patients. At the very least the results are valid for the cohort described here and as such permit us to state our major findings as described above. From a conceptual point of view this study was not designed as a validation study. The aim of this work was not to construct or reconstruct an instrument. The main study question originated from clinical interest in finding a valid instrument measuring cognitive and mental deficits after major trauma. In this context, we tested the QOLIBRI in comparison to other measures of outcome with the objective of obtaining a more specific instrument to measure such deficits. But contrary to expectations we found that mental and cognitive deficits measured with the QOLIBRI were not specific to TBI patients. There may be two main reasons for this: (1) the QOLIBRI does not measure specific mental and cognitive deficits or (2) major trauma per se may be so traumatizing that mental deficits may result independently from the type and severity of injury. Due to missing normative data or neuropsychological examination of patients, it cannot be conclusively decided at this time whether the QOLIBRI should indeed be improved for better sensitivity with regard to TBI-specific outcome residuals or not. Investigators interested in this topic should combine future questionnaire interviews with a standard clinical examination of individuals to achieve such benchmarking [10]. For this prospective investigation no standard evaluation of pretrauma illness or comorbidity of patients was undertaken. We cannot comment on scores other than those investigated in this study [56]. In addition, evaluations in larger cohorts of major trauma patients and with differing definitions of TBI have to be undertaken.

## Conclusions

Our results revealed cognitive deficits following major trauma independently of whether patients sustained major brain injury or not. With the objective of detecting possible mental deficits, this finding supports the general application of the QOLIBRI and, above all, its cognitive subscore for outcome measurement of severely injured patients. In a next step, objective neuropsychological tests should further validate our approach using patients’ self rating data. Future investigations may additionally reveal the potential benefits of an instrument that could be used directly after the accident, prior to hospital discharge, and at the beginning and end of the rehabilitation programme. This instrument might include more objective questions in the mental dimensions such as ability to think, reason and concentrate, would enhance long-term comparison capabilities, and provide documentation as a progress indicator for both physician and patient [39].

## Abbreviations

AIS: Abbreviated injury scale
EQ-5D: EuroQoL five dimensions questionnaire
GCS: Glasgow coma scale
GOS: Glasgow outcome scale
HRQoL: Health-related quality of life
ISS: Injury severity score
MCS: Mental sum componentof SF-36
NISS: New injury severity score
Q, QOLIBRI: Quality of life after brain injury
RISC: Revised injury severity classification
SAPS II: Simplified acute physiology score II
SD: Standard deviation
SF-36: Trauma medical outcomes study Short Form-36
TBI: Traumatic brain injury
TOP: Trauma outcome profile
VAS: Visual analogue scale
